# News and Coming Events

**Published:** 1907-04-20

**Authors:** 


### NEWS AND COMING EVENTS.

Mr. Pearson Gregory has promised £7,000 towards the Grantham Hospital Endowment Fund.

The Exeter Hospital Saturday Fund has been dissolved, owing to the lack of support which it has received during the last two years.

The annual dinner of the French Dispensary will be held at the Hotel Cecil on Saturday night, April 30. The French Ambassador will occupy the chair.

At the annual meeting of the Littlehampton and District Hospital, the investment of £200 was sanctioned, as the nucleus of an extension building fund.

Four wards comprising 64 beds of the Royal Victoria Hospital, Belfast, are standing empty at present, owing to want of funds.

Sir Gilbert Greenall has been elected president of the Warrington Infirmary on the retirement of Mr. Parr, who has served as president of the institution for more than twenty years.

The Sixteenth International Medical Congress will be held at Budapest from August 2 to September 4, 1909. The General Secretary of the Congress is Professor E. Grosz, M.D., 8 Esterhazy-utcza, 7 Budapest, Hungary.

The annual report of the Hereford General Hospital shows that during the past year careful inquiry has been made into the circumstances of out-patients. Several small improvements have been effected in the wards and buildings.

The *Glasgow News* People's Shilling Fund in aid of the local Children's Hospital is making steady progress, and there seems every possibility that the £10,000 desired will be forthcoming. A noteworthy feature in the subscription list is the number of sixpenny and shilling donations.

"Taking the average for the whole year," says the report of the Royal Boscombe and West Hants Hospital, "there were 36.6 beds occupied every night. This means that on many occasions couches and mattresses on the floor were in use to accommodate urgent cases, while the balconies relieved the pressure on space in the wards. . . . The hospital must continue to work at high pressure until its present accommodation is extended."

Sheffield is badly off for hospital accommodation. It has at present only one hospital bed for every 831 head of population. It is therefore proposed to extend the Royal Hospital, at a cost of £30,000. An appeal, signed by the president, the Duke of Norfolk, has had a generous response. One contributor has offered £5,000 for the erection of a country annex, and a member of the committee has subscribed £500.

A sanatorium is to be built at Salterley Grange, an estate on the Cotswold Hills, near Cheltenham. The building will accommodate some 40 patients. The estate is a charming spot in itself, and it is in the midst of beautiful scenery, the Cotswold uplands in this district being particularly well wooded, and on it stands the famous tree known as the "Salterley Oak," supposed to be the oldest tree of the kind in Gloucestershire, local tradition giving it the age of 2,000 years.

The memorial stone of the new buildings of the Glasgow Royal Infirmary will be laid on April 24 by H.R.H. the Prince of Wales.

Madame George Speyer, of Frankfort, Germany, has given £150,000 towards a fund for the "advancement of scientific research."

Messrs. J. S. Fry and Sons, Limited, makers to H.M. the King, have been appointed by special Royal Warrant manufacturers of chocolate and cocoa to their Majesties the King and Queen of Spain.

During the year 1908 the Royal Dental Hospital, Leicester Square, will complete the fiftieth year of its existence, and a sub-committee has been appointed to consider and report upon the advisability of issuing a special appeal for funds during the jubilee year.

Lord Kinnaird will preside at the annual meeting of the Zenana Bible and Medical Mission, which will be held in Exeter Lower Hall on Friday, April 26, at 3 p.m. Among the speakers at the meeting will be the Hon. G. Kinnaird, who visited the hospitals and stations of the society last year.

His Royal Highness the Duke of Connaught and Strathearn has graciously consented to preside at the festival dinner which is to be held at the Princes Restaurant on Friday, May 10, in aid of the funds of the Middlesex Hospital. This dinner, it will be remembered, was to have been held last year, but, owing to unforeseen circumstances, suffered postponement.

H.R.H. the Duchess of Albany will lay the memorial stone of the new Infants' Hospital, Vincent Square, Westminster, on Thursday, May 2, at 5 p.m. This hospital is the first, and, at present, the only hospital in England exclusively devoted to the scientific treatment of the malnutrition of infants. It does not deal with surgical or acute cases of illness. The new hospital will contain 50 cots.

On Monday afternoon the Prince and Princess of Wales visited the Royal Hospital at Richmond and opened the new "Swan" eye wards. The wards were built by Mrs. Benjamin Bousfield Swan, of Teddington, in memory of her late husband. They consist of a male and female division, together accommodating twelve patients, with attached operating theatre and side rooms. The extensions have cost £4,000, and Mrs. Swan has also given a further sum of £2,000 as the nucleus of an endowment fund. Before the opening ceremony, the Princess of Wales received a number of purses containing contributions towards the maintenance fund, ranging from five guineas to £1,000 given by Sir Max Waechter.

H.R.H. the Prince of Wales will open, on Tuesday, May 7 next, at four o'clock, the important extensions at Tottenham Hospital which have recently been carried out at a cost of £20,000. The extension of the hospital has been undertaken as an absolute necessity owing to the large and thickly populated district which it serves. Her Royal Highness, the Princess of Wales, has kindly consented to receive purses containing £5 or upwards, in aid of the funds, and it is hoped that these, together with a list of special contributions which will be announced at the opening ceremony, and the collection taken upon the occasion, will materially help in reducing the debt of £8,000 which has been accumulating during the past six years.

